# Supplementary material for: Genome-wide unique insertion sequences among five Brucella species and demonstration of differential identification of Brucella by multiplex PCR assay
Source: Sci Rep. 2020 Apr 14;10:6368. doi: 10.1038/s41598-020-62472-3 (PMC7156498; doi:10.1038/s41598-020-62472-3)
Supplement: Supplementary file 1 — Supplementary Information. [file 41598_2020_62472_MOESM1_ESM.docx]

**Genome-wide unique insertion sequences among five *Brucella* species and demonstration of differential identification of *Brucella* by multiplex PCR assay**

**Authors**

**Soumya Paul^1^, Bhavani Venkataswamachari Peddayelachagiri^1^, Madhurjya Gogoi^2^, Sowmya Nagaraj^1^, Shylaja Ramlal^1^, Balakrishna Konduru^1^, Harsh V Batra^*1^**

^1^Microbiology Division, Defence Food Research Laboratory, Mysore, Karnataka, India.

^2^Centre for Biotechnology and Bioinformatics, Dibrugarh University, Dibrugarh 786004, Assam, India.

**Corresponding Author:** Dr. Harsh Vardhan Batra (Retired Director), Food Microbiology Division

Defence Food Research Laboratory, E-mail: [drbatrahvardhan@gmail.com](mailto:drbatrahvardhan@gmail.com), Phone:+0091-98440 62686, Fax: 0821-2341923

| ***Brucella* species** | **Chromosome I** | | | **Chromosome II** | | |
| --- | --- | --- | --- | --- | --- | --- |
|  | **Size**  **(Mbp)** | **Total No. of fragments** | **No. of fragments selected** | **Size**  **(Mbp)** | **Total No. of fragments** | **No. of fragments selected** |
| *Brucella abortus* A13334 | 2.123 | 2124 | 4 | 1.162 | 1163 | 21 |
| *Brucella melitensis* ATCC 23457 | 2.125 | 2126 | 22 | 1.185 | 1186 | 5 |
| *Brucella ovis* ATCC 25840 | 2.111 | 2112 | 21 | 1.164 | 1165 | 65 |
| *Brucella suis* 1330 | 1.923 | 1924 | 13 | 1.400 | 1401 | 24 |
| *Brucella canis* ATCC 23365 | 2.105 | 2106 | 2 | 1.206 | 1207 | 1 |

**Supplementary Table 1. Whole genome sequences of *Brucella* species used in this study for *in silico* mining of *Brucella* species-specific targets**

| **Accession Number** | **Sequence ID** |
| --- | --- |
| **MG888755** | *Brucella abortus* strain S19 hypothetical protein gene |
| **MG888756** | *Brucella melitensis* strain 16M hypothetical protein gene |
| **MG888757** | *Brucella suis* 1330 hypothetical protein gene |
| **MG888758** | *Brucella canis* strain ATCC 23365 hypothetical protein gene |
| **MG888759** | *Brucella ovis* ATCC 25840 hypothetical protein gene |
| **MG888760** | *Brucella melitensis* strain DFRL1M hypothetical protein and porin family protein genes |
| **MG888761** | *Brucella abortus* strain DFRL1A hypothetical protein and porin family protein genes |
| **MG888762** | *Brucella suis* strain DFRL1S hypothetical protein and porin family protein genes |
| **MG888763** | *Brucella canis* strain ATCC 23365 hypothetical protein and porin family protein genes |
| **MG888764** | *Brucella ovis* ATCC 25840 hypothetical protein and porin family protein genes |
| **MG888765** | *Brucella ovis* ATCC 25840 phosphoglycerate kinase gene |
| **MG888766** | *Brucella abortus* strain DFRL2A phosphoglycerate kinase gene |
| **MG888767** | *Brucella melitensis* strain DFRL2M phosphoglycerate kinase gene |
| **MG888768** | *Brucella suis* strain DFRL2S phosphoglycerate kinase gene |
| **MG888769** | *Brucella canis* strain ATCC 23365 phosphoglycerate kinase gene |
| **MG888770** | *Brucella suis* strain DFRL3S flagellar protein export ATPase gene |
| **MG888771** | *Brucella abortus* strain DFRL3A flagellar protein export ATPase gene |
| **MG888772** | *Brucella melitensis* strain DFRL3M flagellar protein export ATPase gene |
| **MG888773** | *Brucella ovis* ATCC 25840 flagellar protein export ATPase gene |
| **MG888774** | *Brucella canis* strain ATCC 23365 flagellar protein export ATPase gene |
| **MG888775** | *Brucella canis* strain ATCC 23365 GNAT family N-acetyltransferase gene |
| **MG888776** | *Brucella abortus* strain DFRL4A GNAT family N-acetyltransferase gene |
| **MG888777** | *Brucella melitensis* strain DFRL4M GNAT family N-acetyltransferase gene |
| **MG888778** | *Brucella suis* strain DFRL4S GNAT family N-acetyltransferase gene |
| **MG888779** | *Brucella ovis* ATCC 25840 GNAT family N-acetyltransferase gene |

**Supplementary Table 2: List of accession numbers nucleotide sequences obtained during development of multiplex PCR**

**Figure Legends**

**Supplementary Figure 1:** Multiple sequence alignment of *B. melitensis* (NC_007618)*, B. ovis* (NC_009505)*, B. suis* (CP002997) and *B. canis* (CP000872) nucleotide sequences possessing unique intergenic nucleotide sequence with that of *B. abortus* (NC_016795) lacking the unique intergenic nucleotide sequence.

**Supplementary Figure 2:** Multiple sequence alignment of *B. abortus* (NC_016795)*, B. ovis* (NC_009505)*, B. suis* (CP002997) and *B. canis* (CP000872) nucleotide sequences possessing unique intergenic nucleotide sequence with that of *B. melitensis* (NC_007618) lacking the unique intergenic nucleotide sequence.

**Supplementary Figure 3:** Multiple sequence alignment of *B. abortus* (NC_016795)*, B. melitensis* (NC_007618)*, B. ovis* (NC_009505) and *B. canis* (CP000872) nucleotide sequences possessing unique intergenic nucleotide sequence with that of *B. suis* (CP002997) lacking the unique intergenic nucleotide sequence.

**Supplementary Figure 4:** Multiple sequence alignment of *B. abortus* (NC_016795)*, B. melitensis* (NC_007618)*, B. suis* (CP002997) and *B. canis* (CP000872) nucleotide sequences possessing unique intergenic nucleotide sequence with that of *B. ovis* (NC_009505) lacking the unique intergenic nucleotide sequence.

**Supplementary Figure 5:** Multiple sequence alignment of *B. abortus* (NC_016795)*, B. melitensis* (NC_007618)*, B. ovis* (NC_009505) and *B. suis* (CP002997) nucleotide sequences possessing unique intergenic nucleotide sequence with that of *B. canis* (CP000872) lacking the unique intergenic nucleotide sequence.

**Supplementary Figure 6:** Schematic representation of the insertional sequence in (A) *Brucella abortus* specific fragment and (B) rescue fragment in *B. melitensis, B. suis, B. canis* and *B. ovis*

**Supplementary Figure 7:** Schematic representation of the insertional sequence in (A) *Brucella melitensis* specific fragment and (B) rescue fragment in *B. abortus, B. suis, B. canis* and *B. ovis*

**Supplementary Figure 8:** Schematic representation of the insertional sequence in (A) *Brucella suis* specific fragment and (B) rescue fragment in *B. abortus, B. melitensis, B. canis* and *B. ovis*

**Supplementary Figure 9:** Schematic representation of the insertional sequence in (A) *Brucella canis* specific fragment and (B) rescue fragment in *B. abortus, B. melitensis, B. suis* and *B. ovis*

**Supplementary Figure 10:** Schematic representation of the insertional sequence in (A) *Brucella ovis* specific fragment and (B) rescue fragment in *B. abortus, B. melitensis, B. suis* and *B. canis*
