# Supplementary material for: Genome-wide unique insertion sequences among five Brucella species and demonstration of differential identification of Brucella by multiplex PCR assay
Source: Sci Rep. 2020 Apr 14;10:6368. doi: 10.1038/s41598-020-62472-3 (PMC7156498; doi:10.1038/s41598-020-62472-3)
Supplement: Supplementary file 2 — Supplementary Dataset. [file 41598_2020_62472_MOESM2_ESM.zip › SREP-19-32359_Revised_Manuscript_10.01.2020.docx]

**Genome-wide unique insertion sequences among five *Brucella* species and demonstration of differential identification of *Brucella* by multiplex PCR assay**

**Authors**

**Soumya Paul^1^, Bhavani Venkataswamachari Peddayelachagiri^1^, Madurjya Gogoi^2^, Sowmya Nagaraj^1^, Shylaja Ramlal^1^, Balakrishna Konduru^1^, Harsh V Batra^*1^**

^1^Microbiology Division, Defence Food Research Laboratory, Mysore, Karnataka, India.

^2^Centre for Biotechnology and Bioinformatics, Dibrugarh University, Dibrugarh 786004, Assam, India.

**Corresponding Author:** Dr. Harsh Vardhan Batra (Retired Director), Food Microbiology Division

Defence Food Research Laboratory, E-mail: [drbatrahvardhan@gmail.com](mailto:drbatrahvardhan@gmail.com), Phone:+0091-98440 62686, Fax: 0821-2341923

**Abstract**

Brucellosis is a neglected zoonotic disease caused by alpha proteobacterial genus *Brucella* comprising of facultative intracellular pathogenic species that can infect both animals and humans. In this study, we aimed to identify genome-wide unique insertion sequence (IS) elements among *Brucella abortus, B. melitensis, B. ovis, B. suis* and *B. canis* for use in species differentiation by conducting an intensive *in silico*-based comparative genomic analysis. As a result, 25, 27, 37, 86 and 3 unique ISs were identified respectively and they had a striking pattern of distribution among them*.* To explain, a particular IS would be present in 4 species with 100% identity whereas completely absent in the 5^th^ species. However, flanking regions of that IS element would be highly identical and conserved in all 5 species. Species-specific primers designed on these flanking conserved regions resulted in two different amplicons grouping the species into two: one that possesses IS and the other that lacks it. Seeking for species-specific amplicon size for particular species was sufficient to identify it irrespective of biovar. A multiplex PCR developed using these primers resulted in successful differentiation of the 5 species irrespective of biovars with significant specificity and sensitivity when examined on clinical samples.

**Keywords**

Insertional sequences; *Brucella*; brucellosis; multiplex PCR; internal amplification control.

**Introduction**

*Brucella*, the etiological agent of a classical zoonotic disease named brucellosis, is an alpha-2 proteobacteria. The genus *Brucella* includes twelve species: *Brucella abortus, B. melitensis, B. suis, B. canis, B. ovis, B. neotomae, B. microti, B. pinnipedialis, B. ceti*, *B. inopinata*, *B. papionis,* and *B. vulpis* ^[1]^*.* These species are aerobic, facultative, intracellular pathogens with specific host preferences for infection and are classified based on diversity in terms of host specificity, phenotypic traits, and pathogenicity. Of these species, *B. abortus*, *B. melitensis* and *B. suis* are further classified into 8 (1-7, 9), 3 (1-3) and 5 (1-5) biovars, respectively. They exhibit a high degree (99%) of genetic homogeneity, as demonstrated by comparative whole genome analysis ^[2]^.

Although genetic polymorphisms in *Brucella* are very limited, they have been satisfactorily used in discriminating and genotyping *Brucella* species ^[3-9]^. Insertion sequences (ISs) are one such genetic element that are reasonably useful in discriminating *Brucella* species with few limitations. Although the role of IS elements and the reason for their occurrence are highly debated, their distribution has impacted genome evolution.

*IS711* is a characteristic IS element among *Brucella* species. The variable copy numbers and positions of IS elements among species have been reported to render species-associated polymorphisms that have shown promising application in *Brucella* molecular typing and identification. *B. melitensis* and *B. suis* contain seven complete *IS711* copies ^[10]^. *B. abortus* carries six complete copies and one truncated *IS711* copy ^[11]^, *B. ceti* and *B. pinnipedialis* carry more than 20 copies ^[12, 13]^ and *B. ovis* carries 38 copies ^[14]^. In the 1990s, *IS711* was shown to be highly stable, and only under experimental conditions was this IS element transposed in *B. ovis* and *B. pinnipedialis* but not in *B. melitensis* or *B. abortus* ^[10]^. *IS711* is characteristic of *B. abortus* and is present in variable numbers and positions but is always present within a given species ^[15]^. The stability of *IS711* in the genome was exploited in the development of the famous AMOS multiplex PCR assay to differentiate *B. abortus*, *B. melitensis*, *B. ovis* and *B. suis* from each other ^[16]^. Thus, the stability of *IS711* was not only relevant for *Brucella* typification: its mobility was implicated in the generation of genetic diversity and speciation, as shown by the distribution of *IS711* among *Brucella* species. However, in 2011, Mancilla and coworkers reported the mobility of *IS711* in *B. abortus* field strains under natural conditions, causing polymorphism within the strains of *B. abortus*. The study reported *B. abortus* strains isolated from cattle carrying seven copies of complete *IS711* instead of six copies, and this new copy was found to be located in a completely new locus that was not previously reported to harbor *IS711*. The reason for the presence of this one additional copy of IS was predicted to be replicative transposition within the genome ^[15]^. However, in another study, novel insertion of *IS711* within the *Omp31* gene of an atypical *B. ovis* isolate recovered from an infected ram in Hungary was encountered and was not correctly identified in the Bruce-ladder multiplex PCR assay ^[17]^. Based on these new case reports, the fact that the most acclaimed IS element, *IS711*, could be a transposon element in *Brucella* species was established, and its reliability in differentiating *Brucella* species was weakened as it demonstrated polymorphism within the strains of a species. With this, the diagnostic application of *IS711* reveals the need for a more improved approach to decipher polymorphisms among *Brucella* species that is even more unique and reliable in differentiating species, even at the biovar level, if possible.

In the current study, we describe a novel *in silico-*based comparative genomic analysis approach to decipher species-associated unique and precise DNA block distributions among 5 species of *Brucella,* namely, *B. abortus,* *B. melitensis, B. ovis, B. suis* and *B. canis* irrespective of biovars. *B. abortus*, *B. melitensis*, *B. suis* and *B. canis* were selected as representative zoonotic species of *Brucella*, and *B. ovis* was selected as a representative species of non-zoonotic *Brucella*. Due to the unavailability of the remaining 7 species in our repository, they were not included in this study. Preliminary *in silico* characterization of the conserved flanking regions indicated that the DNA blocks were inserted in transposition sites, indicating that the DNA blocks were actually IS elements. This was achieved by conducting an intensive *in silico*-based comparative genomic analysis of *Brucella* species using the Perl language and the vast data available online on whole genome sequences of *Brucella* species. The distribution of the precise stretch of DNA blocks among the five species was unique, as a stretch of nucleotide sequence was embedded within a conserved region of the genome in four of five species but absent in the fifth species. However, the conserved flanking regions (where the primer pairs are located) demonstrated 100% homology in all 5 species. Furthermore, these highly specific nucleotide sequences were evaluated for their ability to detect targets, and a novel multiplex PCR was developed wherein the 5 species were differentiated irrespective of biovars.

**Materials and Methods**

**Bacterial strains**

The *Brucella* strains (Table 1) were clinical isolates preserved in the repository of the Defence Food Research Laboratory (DFRL). The clinical strains recovered from infected animals and patients were identified by biochemical tests and DNA sequencing. Reference strains of *Pseudomonas aeruginosa*, *P.* *azotogenesis, Aeromonas hydrophila*, *Proteus mirabilis*, *Citrobacter* *freundii* and *Yersinia enterocolitica* were used as negative controls in the specificity study. All bacterial strains were aseptically recovered from glycerol stocks and grown in trypticase soy broth (TSB) overnight at 37 °C. Genomic DNA from harvested cultures was prepared using a QIAamp DNA Mini Kit (Qiagen, Germany) according to the manufacturer’s instructions. DNA samples were determined using a Nanodrop^TM^ 1000 spectrophotometer (Thermo Scientific, MA, USA), stored at -20 °C and used in PCR.

**Biosafety procedures**

All procedures using *Brucella* strains were performed in a biosafety level 3 laboratory. Procedures for culture of non-*Brucella* species and DNA extraction were conducted in a class II type A2 biological cabinet (Thermo Scientific, USA).

**Genomic analysis of *Brucella* species**

Whole genome sequences (WGSs) of *B. abortus* A13334, *B. melitensis* ATCC 23457, *B. ovis* ATCC 25840, *B. suis* 1330, *B. suis* ATCC 23445, *B. suis* VBI22 and *B. canis* ATCC 23365 were used for *in silico* mining of *Brucella* species-specific targets (Supplementary Table 1). Briefly, the whole genome sequences of the selected *Brucella* strains were cut into fragments of 1000 bp by implementation of a script written in the Perl programming language. The nucleotide fragments of each species (for instance, *B. abortus*) were aligned against the genomic sequences of all other biovars within the species using the BLASTN program. Nucleotide fragments that matched all biovars with an E-value less than 10^-200^ were identified as highly specific targets for *B. abortus* irrespective of biovars (Figure 1). The procedure was repeated with WGSs of *B. melitensis*, *B. suis*, *B. canis* and *B. ovis*. Thus, the conserved DNA fragments from each species of *Brucella* were determined and further aligned against the genomic sequences of the other four species to evaluate their specificity.

**Analysis of *Brucella* species-specific DNA fragments**

The determined species-specific fragments of *Brucella* were analyzed for conservation and variation by multiple sequence alignment using the CLUSTALW tool available on the EMBL-EBI website (http://www.ebi.ac.uk/Tools/msa/clustalw2/) and by BLAST analysis against the vast WGS database available in the National Center for Biotechnology Information database (http://www.ncbi.nlm.nih. gov/) and the Broad Institute (http://www.broadinstitute.org). The respective protein(s) or peptide(s) encoded by each of the species-specific fragments of *Brucella* were determined using Gene Runner 3.0 software (http://www.generunner.net/). Thus, the determined protein(s) or peptide(s) sequences were analyzed by BLASTP analysis of the National Center for Biotechnology Information (http://www.ncbi.nlm.nih.gov/). Protein sequence features, including the identification of conserved domains, protein superfamily and further protein classification, were performed using the National Center for Biotechnology Information.

**Primer design**

Primers for specific PCR amplification of *Brucella* species-specific fragments were designed using Gene Runner 3.0 software (http://www.generunner.net/). The primers were designed to have T_m_ values ranging from 52-68°C and ΔG values for primer duplex below -10 kcal/mol and not to form hairpin loops or primer dimers. The strategy of designing primers is illustrated in Figure 2. The primers were designed with the intent of obtaining specific yet easily distinguishable and interpretable amplicons under identical PCR conditions and were thus usable in the development of a multiplex PCR system. Designed primers were analyzed for specificity by using the BLASTN program prior to synthesis. To prevent false negative results due to the interference of inhibitory substances in test samples, competitive internal amplification control (IAC) was generated using the pUC19 plasmid as the target. The IAC primers were designed such that they had 5’ overhanging ends identical to the primer sequences of the *B. abortus-*specific primer pair and 3’ ends complementary to the pUC19 plasmid, amplifying 808 bp of the pUC19 plasmid and yielding a product of 847 bp (inclusive of the 5’ *B. abortus*-specific flanking regions of the primer pair). The sequences of the designed primers are presented in Table 2. All primers were synthesized by Eurofins India, India.

**Monoplex PCR**

For initial standardization of the detection system, monoplex PCR was performed using the primer sets and respective genomic DNA as template. Each 25 µL PCR contained the following: 1 U of *Taq* polymerase, 250 µM each deoxynucleotide triphosphate, 1x PCR buffer (including 1.5 mM MgCl_2_), 10 pmol of each oligonucleotide primer, and 25 ng of template DNA. Thermal cycling was carried out in an Eppendorf thermal cycler (Eppendorf, USA) for 35 cycles of 30 s at 94 °C, annealing for 30 s at 56 °C, and extension at 72 °C for 1 min, with a final 8 min extension at 72 °C. Approximately 2 µl of each PCR product was visualized by agarose gel electrophoresis. The template DNA of each species was used with its respective specific primer in each monoplex PCR.

**IAC detection limit**

The 847 bp IAC amplicon amplified using chimeric oligonucleotide primers was purified using a QIAquick PCR Purification Kit (Qiagen, Germany) and further subjected to 1000-fold serial dilutions from 1:1000 to 1:10000. From each dilution, 1 µl of the amplicon was used as template along with 25 ng of *B. abortus* gDNA and PCR-amplified in a single reaction using *B. abortus-*specific primers. The PCR components and amplification conditions were the same as described in the previous methods section. The lowest dilution of IAC DNA resulting in an amplicon of the same intensity as *B. abortus* DNA was selected for standardization of multiplex PCR.

**Multiplex PCR assay**

Twenty-five nanograms of genomic DNA from each *Brucella* species, namely, *B. abortus, B. melitensis, B. canis, B. suis and B. ovis*, along with a 1:9000 dilution of IAC DNA, was subjected to each multiplex PCR in a 50 µl reaction mixture containing 1x PCR buffer, 1.5 mM MgCl_2_, 200 mM each deoxynucleotide triphosphate, 10 pmol of each oligonucleotide primer and 1 U of *Taq* polymerase (Sigma Aldrich, India). PCR amplification was carried out as follows: initial denaturation at 94 °C for 10 min; 30 cycles of 94 °C for 1 min, 56 °C for 1 min and 72 °C for 1.30 min; and a final elongation step at 72 °C for 8 min. Amplicons were loaded onto a 2% agarose gel containing ethidium bromide that was run at 100 V for 1 h. To evaluate the reproducibility of the developed assay, the PCR was repeated three times in three different thermal cyclers: Mastercycler Gradient (Eppendorf, USA), C1000 Touch^TM^ (Biorad, USA) and Techne TC-3000G (Techne, UK).

**Validation of the multiplex PCR assay for specificity**

To evaluate the specificity of the multiplex PCR assay, purified genomic DNA isolated from all bacteria listed in Table 1 was employed. Nucleic acid was isolated using the QIAamp DNA Mini Kit (Qiagen) by following the manufacturer’s instructions. Ten microliters of the eluted DNA was used to perform a multiplex PCR assay.

**Statistical analysis**

Descriptive statistics were performed to evaluate the specificity of the multiplex PCR assay. Reproducibility of the results for multiplex PCR optimization and overall specificity percentage was examined by repeating the blind experiments three times. Kappa statistical analysis was used to measure false negative results. Kappa estimates were defined as follows: values < 0. 2 imply poor agreement, values from 0.2-0.6 imply fair to moderate, and values > 0. 6 imply good or very good agreement ^[18]^.

**Data access**

The respective specific and rescue nucleotide sequences of the *B. abortus, B. melitensis, B. canis, B. suis and B. ovis* reference strains used in this study were submitted to GenBank under the **accession numbers MG888755 to MG888779** (Supplementary Table 2).

**Results**

**Mining of *Brucella* species-specific detection targets**

On average, 3405 fragments were obtained after dividing the genomes of *B. abortus* A13334, *B. melitensis* ATCC 23457, *B. ovis* ATCC 25840, *B. suis* 1330, *B. suis* ATCC 23445, *B. suis* VBI22 and *B. canis* ATCC 23365 into 1000 bp fragments. The derived fragments were analyzed by BLASTN analysis with genome sequences of *Brucella* strains available on the NCBI database, and a majority of the fragments showed high sequence identity (E values < 10^-200^). These conserved sequences from each species of *Brucella* were further aligned against the genomic sequences of the remaining four species to evaluate their uniqueness. This analysis revealed 25, 27, 37, 86 and 3 conserved nucleotide fragments in *B. abortus*, *B. melitensis*, *B. suis*, *B. ovis* and *B. canis*, respectively, and the fragments had a striking pattern of distribution among them*.* Specifically, a stretch of nucleotide sequence would be present in 4 species with 100% identity but completely absent in the 5^th^ species. However, the flanking regions of that stretch of nucleotide sequence would be highly identical in all 5 species. For instance, a 1000 bp fragment of *B. abortus* was present in *B. melitensis*, *B. suis*, *B. ovis* and *B. canis* with an additional 324 bp unique interrupting intergenic nucleotide stretch within the fragment (that was strikingly absent in *B. abortus*), making the fragment 1324 bp in length. Identical patterns were observed in the remaining four species of *Brucella*. Supplementary Figures 1 to 5 (*B. abortus*, *B. melitensis*, *B. suis*, *B. ovis* and *B. canis*) represent multiple sequence alignments of randomly selected unique intergenic and/or intragenic sequences, one each from the 5 *Brucella* species of interest.

***Brucella abortus* specific fragment**

The *B. abortus-*specific fragment of 1154 bp and its respective rescue fragment of 1478 bp from the four other *Brucella* species of interest were found to be comprised of an unknown conserved region 299 bp from the forward primer position followed by a conserved hypothetical protein encoding region in an inverted position (Supplementary Figure 6). Interestingly, no features of insertional sequences, including direct repeats, terminal inverted repeats, translational frame shifting or translational termination, were observed.

***Brucella melitensis-*specific fragment**

The *B. melitensis-*specific fragment of 745 bp was comprised of a C-terminal portion (including a stop codon) of an unknown gene encoding a hypothetical protein, an unannotated region 223 bp length and a portion (366 bp) of the gene encoding Omp31, including its open reading frame. In the case of its respective rescue fragment of 977 bp from the other four *Brucella* species, an insertion of a truncated stretch of the ORF of a gene encoding the N-terminal portion of Omp31b within the ORF of the *Omp31* gene was observed. Integration of this insertional sequence was accompanied by the duplication of a 14 bp direct target repeat upstream of the insertional sequence (Supplementary Figure 7).

***Brucella suis-*specific fragment**

The *B. suis-*specific fragment of 290 bp and its respective rescue fragment of 383 bp from the four other *Brucella* species of interest were identified as a portion of the gene encoding flagellum-specific ATP synthase, *fliI*, belonging to the flagellum-specific ATPase/type III secretory pathway virulence-related protein. ATPases of this group are responsible for the export of flagellum and virulence-related proteins. The bacterial flagellar motor is similar to the F0F1-ATPase, in that they both are proton-driven rotary molecular devices. However, the main function of the bacterial flagellar motor is to rotate the flagellar filament for cell motility. Intracellular pathogens such as *Salmonella* and *Chlamydia* also have proteins that are similar to the flagellar-specific ATPase but function in the secretion of virulence-related proteins via the type III secretory pathway. The gene was found to be directional within the genome of the *Brucella* species. Similar to the case of *B. abortus-*specific and rescue fragments, no features of insertional sequences, including direct repeats, terminal inverted repeats, translational frame shifting or translational termination, were observed (Supplementary Figure 8).

***Brucella canis-*specific fragment**

The *B. canis-*specific fragment of 224 bp and its respective rescue fragment of 521 bp from four other *Brucella* species of interest were identified to be the structural domain of the acyl-CoA acyltransferase enzyme from GNAT family N-acetyltransferase. This domain has a 3-layer alpha/beta/alpha structure that contains mixed beta-sheets and can be found in proteins including N-acetyl transferase (NAT) family members, aminoglycoside N-acetyltransferases, diamine acetyltransferase 1, autoinducer synthetases such as protein LasI and acyl-homoserinelactone synthase EsaI, the histone acetyltransferase domain of P300/CBP-associating factor PCAF and the catalytic domain of GCN5 histone acetyltransferase. The insertional sequence had a length of 298 bp, and the structural domain of this gene encoding acyl-CoA acyltransferase was also annotated to be a part of this structural domain inserted between flanking direct “AT” repeats at the 128^th^ and 426^th^ nucleotide positions of the rescue fragment (Supplementary Figure 9).

***Brucella ovis-*specific fragment**

The *B. ovis-*specific fragment of 446 bp comprised a C-terminal portion (336 bp) of phosphoglycerate kinase encoded by the *pgk* gene, a 33 bp DNA block encoding transposase, a 9 bp direct repeat, a 35 bp DNA block encoding DNA translocase and a 33 bp DNA sequence encoding the N-terminus of a peptidase. On the other hand, the rescue fragment of the same conserved stretch in the other four *Brucella* species contained a 150 bp DNA block comprising an 87 bp DNA fragment encoding an outer membrane autotransporter barrel domain, 24 bp of an unknown nucleotide sequence and a 39 bp truncated stretch of DNA sequence encoding a portion of a protein belonging to the ABC transporter ATP-binding protein group. The insertion of this 150 bp DNA block was found to be after the direct repeat observed in the specific fragment (Supplementary Figure 10).

**PCR assay optimization**

For initial standardization of the detection system, monoplex PCR was performed using each primer pair and respective genomic DNA as template. The result of each monoplex PCR is represented in Figure 3. Monoplex PCR using *B. abortus*, *B. melitensis*, *B. suis*, *B. ovis* and *B. canis* in the presence of their specific primer pairs yielded amplicons of 1154 bp, 745 bp, 290 bp, 446 bp and 224 bp, respectively. Sequencing of each PCR amplicon revealed the complete identity of the amplified products with their specific genes. When the reaction was performed using genomic DNA from all 5 *Brucella* species in a single PCR test with any one pair of primers as mentioned in the methods section, each primer pair amplified a specific amplicon of a particular size with its respective species and a rescue amplicon with the remaining 4 species that was longer in length when compared to the respective specific amplicons; therefore, the amplicons were grouped into separate group altogether (Figure 4). The rescue amplicons would provide additional information on the presence of other *Brucella* species in the test sample and hence rules out the chance of having nonspecific amplification with other contaminating organisms. For instance, the primer specific for *B. abortus* resulted in a 1154 bp amplicon with *B. abortus* and a 1478 bp amplicon with 4 other species, namely, *B. melitensis, B. suis, B. ovis* and *B. canis.* The multiplex PCR described in this study includes a total of 6 pairs of primers: one pair for each of the 5 *Brucella* species*,* namely, *B. abortus, B. melitensis, B. canis, B. suis and B. ovis*, and one pair for the internal amplification control. In our multiplex PCR assay, each primer pair had the capability to detect a specific *Brucella* species and differentiate it from the other 4 species. This species-dependent differential amplification was noticed in the case of all specific primers, wherein the primer-specific species would result in an amplicon different from the amplicon size yielded by the remaining 4 species.

**Interpretation of a multiplex PCR system**

The multiplex PCR described here includes a total of 6 pairs of primers: one pair for amplification of each of the 5 *Brucella* species, namely, *B. abortus, B. melitensis, B. canis, B. suis and B. ovis*, and one for the internal amplification control (Figure 5). Each pair of *Brucella* species primers mentioned above can yield PCR amplicons for all 5 species, resulting in a specific amplicon of a particular size with its respective species and a rescue amplicon longer than the specific amplicon with the other 4 species, thus differentiating them from the 5^th^ *Brucella* species. For example, the *B. abortus*-specific primer pair results in a 1154 bp amplicon with *B. abortus* and a 1478 bp amplicon with 4 other species, namely, *B. melitensis, B. suis, B. ovis* and *B. canis.* The interpretation of the multiplex PCR assay described here is illustrated in Figure 6. In an instance where multiplex PCR is performed with a test sample contaminated with all 5 *Brucella* species, namely, *B. abortus, B. melitensis, B. suis, B. canis* and *B. ovis*; the result will be as follows: (1) the *B. abortus-*specific primer pair yields an amplicon of 1154 bp with *B. abortus* and 1478 bp with the other 4 species; (2) the *B. melitensis-*specific primer pair yields an amplicon of 745 bp with *B. melitensis* and an amplicon of 977 bp with the other 4 species; (3) the *B. suis-*specific primer pair yields an amplicon of 290 bp with *B. suis* and an amplicon of 383 bp with the other 4 species; (4) the *B. canis-*specific primer pair yields an amplicon of 224 bp with *B. canis* and an amplicon of 521 bp with the other 4 species; (5) the *B. ovis-*specific primer pair yields an amplicon of 446 bp with *B. ovis* and an amplicon of 606 bp with the remaining 4 species; and (6) the IAC-specific primer yields an amplicon of 847 bp with IAC DNA.

**Assay specificity**

The multiplex PCR assay developed was evaluated with 80 *Brucella* reference strains and isolates from humans and animals. The assay demonstrated a high rate of specificity (Table 1) in the case of *B. abortus* and *B. melitensis* irrespective of biovars with respect to their specific primers. All the biovars from these two species gave the same band profile, and the selected species were differentiated by the specific and rescue amplicon profiles. However, in the case of *B. suis*, biovars 3, 4 and 5 resulted in a 383 bp rescue amplicon with *B. suis-*specific primers instead of the expected 290 bp amplicon, making the said biovars incompatible with the proposed strategy and indistinguishable from other species of *Brucella*. The *B. suis* ATCC 23445 strain also produced amplicons 383 bp in length. The *B. canis-*specific primer pair resulted in a rescue amplicon 224 bp in length with *B. abortus* biovar 5 instead of the expected 521 bp amplicon. The *B. ovis-*specific primer pair resulted in a nonspecific amplicon 832 bp in length with *B. abortus* biovar 5 instead of the expected 606 bp amplicon. These exceptional cases were encountered even during the *in silico* analysis of the designed primers for reliability in multiplex PCR. No amplification was recorded with the 6 non-*Brucella* species tested.

**Discussion**

The characteristic feature of bacterial ISs is their capacity to generate mutations in an organism by transposition within the genome. ISs occur in various copy numbers in a particular genome and can move within the genome or even between genomes horizontally, resulting in clonal expansion of species in a genus ^[19]^. Intensive studies with respect to insertion sequences have unraveled genetic features, namely, direct repeats, terminal inverted repeats, translational frame shifting or translational termination, segmental genome duplication and several other features associated with genetic insertion and/or translocation of DNA blocks in bacterial genomes ^[20]^.

The transposition of ISs is generally identified by the presence of short terminal inverted repeats that flank these DNA blocks. Many ISs also carry one or two open reading frames encoding genes that are essential for their transposition. The mechanism of transposition is primarily dependent on catalysts, including transposase and inverted repeats necessary for the binding of transposase, which in turn results in donor DNA cleavage and strand transfer ^[20]^. The presence and transposition of IS elements within and between genomes are associated with gene inactivation, modulating virulence, the expression of neighboring genes, antibiotic resistance and metabolism ^[21]^. Irrespective of IS families, elevated transposition of ISs between genomes serves to amplify both deleterious and advantageous fitness changes, but this accelerates the extinction of elements, particularly if the mean fitness effect of IS-induced mutations is negative. However, transposition bursts create genetic diversity, which occasionally generates advantageous mutations and thus help organisms adapt to new environments ^[22]^. In this line of IS-mediated mutations among prokaryotes, several bacterial models, including *Enterobacteriaceae* members, *Pseudomonas* sp., *Acinetobacter baumannii*, *Lactobacillus* sp., *Burkholderia* sp., *Staphylococcus aureus* and *Vibrio* sp. have been successfully explored and studied. These studies have supported the establishment of a knowledge base on the involvement of ISs in the plasticity and adaptability of bacterial genomes ^[21]^. However, in the case of the genus *Brucella*, very little information is available on the diversity of ISs within the genomes, and only IS*711* has been extensively studied in this respect until now. With this background, we designed our study to explore novel genetic polymorphisms among *Brucella* species that can be exploited to establish the differentiation among them for diagnostic applications.

The distinctively remarkable pattern of insertions and deletions of insertional sequences with precise lengths with the determined target sequences in our study, which was exploited in the development of a novel multiplex PCR assay for use in the diagnosis of brucellosis, encouraged us to understand the mechanism involved in the presence or absence of such precise lengths of IS elements. Our opinion was that one mechanism was possibly involved in the unique pattern of inserting DNA blocks with identical lengths in four different species of *Brucella*, but not one species. To our surprise, the diversity was not limited to the range of complexity observed among each fragment but to the possible mechanisms involved in the transposition of the DNA blocks.

The typical segmental genome duplication observed in the case of the insertion of the *Omp31b* gene fragment within the conserved *Omp31* gene is a feature usually observed among the IS*21* family. Segmental duplications usually occur at more than one site within a given genome with a high level of sequence identity, as confirmed by both *in silico* and *in situ* hybridization experiments, and such patterns are usually observed in the human genome ^[23, 24]^. Such segmental duplications are observed as an indicative sign of evolutionary rearrangement ^[25, 26]^.

In the case of the *B. abortus* and *B. suis* rescue fragments in our study, no features of insertional sequences were observed. A lack of such features is an indication of possible homologous intermolecular or intramolecular recombination that would have occurred between two insertional elements each with different direct repeats. Such recombination usually results in the formation of a hybrid element carrying one direct repeat of each parent. In some cases, formation of adjacent deletions from duplicative intramolecular transpositions can also result in such insertional sequence elements. A single copy of the direct repeat could be located on each of the deletion products in such cases. However, extensive studies are required to confirm the exact mechanism involved in such insertion events on a case-by-case basis throughout the genome.

The generation of short directly repeated sequences flanking the inserted DNA sequences is a general feature of insertional sequence elements. Such insertions are facilitated by the attack of each DNA strand at the target direct repeat site by one of the two transposon ends in a staggered way. Usually, the lengths of such direct repeats range between 2 and 14 bp. In the case of the *B. canis* rescue fragment, the flanking direct repeat was 2 bp in length. Usually, in the case of such direct repeats, the insertion sequence will be generated as a genome duplication of a fixed length, but nevertheless, generation of small variations in the transposition complex is also observed at a low frequency. Latter condition was observed in the rescue fragment of *B. canis* in our study..

The intention of the present work was to develop a detection system for simultaneous and differential identification of 5 *Brucella* species irrespective of biovars and strains based on comparative genomic analysis using the updated database of *Brucella* genomes. The specificity of the intended detection system is crucial for the diagnosis of brucellosis, which solely depends on the target genes. With the comparative whole genomic analysis approach for mining species-specific nucleotide sequences, a remarkable pattern of insertion or deletion of certain length nucleotides was encountered in the genomes of *B. abortus*, *B. melitensis*, *B. suis*, *B. ovis* and *B. canis*. Interestingly, the deciphered sequences were not limited to genes required for virulence or other specialized metabolic functions but also included noncoding sequences.

The distinctively remarkable pattern of insertions and deletions of nucleotide sequences or DNA blocks with particular lengths with the determined target sequences separated them into two groups, one group with deletions and the other 4 groups with insertions, supporting the development of the exemplary detection tool. The unique sequences were differentially present in 4 species but absent in one and were intergenic and/or intragenic in nature, inserting within conserved regions of the *Brucella* genus as detection targets. Species-specific deletions in the case of *Brucella* were reported earlier by Rajashekara and coworkers (2004) based on microarray analysis, and these deletions were found to be highly conserved. Successful exploitation of those deletions in the design of species-specific primers for the differentiation of *B. neotomae* and *B. canis* was described in the previously mentioned study. The primers in that study not only resulted in different amplicon sizes in the cases of *B. neotomae* and *B. canis* but also showed one or completely different PCR amplification bands in the case of the other *Brucella* species screened. Similar differential patterns were derived for specific and differential identification of other *Brucella* species by the exploitation of different deletions. However, as a limitation in that study, biovars within a species showed different banding patterns, making interpretation a difficult and challenging task ^[27]^.

In the current study, irrespective of biovars, in the unique sequence of *B. abortus*, which we selected randomly, to include as a detection target, a deletion of 324 bp was observed between positions 469 and 470 when base count was performed from the hybridizing position of the *B. abortus* forward primer, resulting in a 1154 bp specific amplicon and a 1478 bp rescue amplicons in the other 4 species. Similarly, upon referring to the forward primer hybridizing position as the base count in the case of the other 4 species, the 745 bp specific amplicon of *B. melitensis* had a deletion of 232 bp between positions 422 and 423 compared to the 977 bp rescue amplicons of the other 4 species. The *B. ovis-*specific amplicon (446 bp) had a deletion of 160 bp between the 379^th^ and 380^th^ positions, differentiating it from the 606 bp rescue amplicon. A 93 bp nucleotide stretch was deleted from the 290 bp specific amplicon of *B. suis* between its 109^th^ and 110^th^ nucleotide positions. When compared with the rescue amplicons of *B. canis*, its specific amplicon had a 297 bp deletion from the 129^th^ nucleotide position, making it a 224 bp product.

Five pairs of oligonucleotide primers were designed to exploit the newly identified species-specific nucleotide sequences for the development of a multiplex PCR assay that identifies 5 *Brucella* species in one single reaction. Initially, each species-specific primer was exploited in the development of a monoplex PCR system for each species with specific amplicons of 1154 bp, 745 bp, 446 bp, 290 bp and 224 bp for *B. abortus, B. melitensis, B. ovis, B. suis* and *B. canis,* respectively. However, when the same 5 species-specific primer pairs were employed in a multiplex PCR for the detection of each species, a unique multiplex PCR system resulted. This was because all the primers would anneal to the conserved regions flanking all 5 nucleotide sequences of the *Brucella* genome, resulting in 4 additional amplicons termed “rescue amplicons” along with the species-specific amplicon. Nevertheless, in a multiplex PCR in the presence of all 5 *Brucella* species, an integrative format comprising all the species-specific amplicons was obtained for the identification and differentiation of all 5 species together in a single lane by gel electrophoresis. Furthermore, these primers were employed in the stabilization of conventional monoplex and multiplex PCR systems. To avoid false negative results, competitive internal amplification control (IAC) was also incorporated in the multiplex PCR system with pUC19 plasmid DNA.

The stabilized monoplex and multiplex PCRs were evaluated for specificity with reference strains of the said 5 *Brucella* species as well as non-*Brucella* species. As an outcome, irrespective of biovars, a highly specific PCR-based detection format was developed that could successfully and differentially identify *B. abortus, B. melitensis, B. ovis, B. suis* and *B. canis*. The major advantage of our assay is that the use of any one primer pair was also sufficient to identify *Brucella* at the genus level and species level by seeking specific and/or rescue amplicons irrespective of biovars. Each pair of primers was useful not only as a species-specific marker but also as a genus-specific marker.

The developed multiplex PCR was used to evaluate its applicability in detecting 80 strains of *Brucella* species. The assay successfully identified the 5 target species and differentiated them from each other. This result reflected the unambiguous specificity of the assay and thus its applicability in the diagnosis of brucellosis. However, to examine the robustness of this tool in clinical cases, multiplex PCR assays require extensive validation in several reference laboratories with a large number of clinical samples.

**Acknowledgements**

The first author is thankful to Indian Council of Medical Research (ICMR), India for support. Second author is thankful to Department of Science and Technology (DST), India for support through INSPIRE program.

**This research did not receive any specific grant from funding agencies in the public, commercial, or not-for-profit sectors.**

**Competing interests**

The authors declare no competing interests.

**Ethical statement**

This article does not contain any studies with human participants or animals performed by any of the authors

**References**

1. El-Sayed, A. & Awad, W. Brucellosis: Evolution and expected comeback. *Int. J. Vet. Sci. Med*. **6**, S31-S35 (2018).
2. Scholz, H. & Vergnaud, G. Molecular characterisation of *Brucella* species. *Rev. Sci. Tech*. *OIE* **32**, 149-162 (2013).
3. Garcia-Yoldi, D. Multiplex PCR assay for the identification and differentiation of all *Brucella* species and the vaccine strains *Brucella abortus* S19 and RB51 and *Brucella melitensis* Rev1. *Clin. Chem.* **52**, 779-781 (2006).
4. Marianelli, C., Petrucca, A., Pasquali, P., Ciuchini, F., Papadopoulou, S. & Cipriani, P. Use of MLVA-16 typing to trace the source of a laboratory-acquired *Brucella* infection. *J. Hosp. Infect*. **68**, 274-276 (2008).
5. Kattar, M. M. *et al*. Evaluation of a multilocus variable-number tandem-repeat analysis scheme for typing human *Brucella* isolates in a region of brucellosis endemicity. *J. Clin. Microbiol*. **46**, 3935-3940 (2008).
6. Huber, B., Scholz, H. C., Lucero, N. & Busse, H.J. Development of a PCR assay for typing and subtyping of *Brucella* species*. Int. J. Med. Microbiol*. **299**, 563-573 (2009).
7. Valdezate, S., Navarro, A., Villalon, P., Carrasco, G. & Saez-Nieto, J. A. Epidemiological and phylogenetic analysis of Spanish human *Brucella melitensis* strains by multiple-locus variable-number tandem-repeat typing, hypervariable octameric oligonucleotide fingerprinting, and *rpoB* typing. *J. Clin. Microbiol.* **48**, 2734-2740 (2010).
8. Lopez-Goni, I. *et al*. Evaluation of a multiplex PCR assay (Bruce-ladder) for molecular typing of all *Brucella* species, including the vaccine strains. *J. Clin. Microbiol.* **46**, 3484-3487 (2008).
9. Ferreira, A. C. *et al*. MLVA16 typing of Portuguese human and animal *Brucella melitensis* and *Brucella abortus* isolates. *PLoS ONE.* 7, e42514 (2012).
10. Ocampo-Sosa, A. A., Agüero-Balbín, J. & García-Lobo, J. M. Development of a new PCR assay to identify *Brucella abortus* biovars 5, 6 and 9 and the new subgroup 3b of biovar 3. *Vet. Microbiol.* **110**, 41-51 (2005).
11. Halling, S. M., et al. Completion of the genome sequence of *Brucella abortus* and comparison to the highly similar genomes of *Brucella melitensis* and *Brucella suis*. *J. Bacteriol.* **187**, 2715-2726 (2005).
12. Bricker, B. J., Ewalt D. R., MacMillan A. P., Foster G., & Brew S. Molecular characterization of *Brucella* strains isolated from marine mammals. *J. Clin. Microbiol.* **38**,1258–1262 (2005).
13. Zygmunt, M. S., Maquart, M., Bernardet, N., Doublet, B. & Cloeckaert, A. Novel IS711-specific chromosomal locations useful for identification and classification of marine mammal *Brucella* strains. *J. Clin. Microbiol.* **48**, 3765-3769 (2010).
14. Tsolis, R. M. *et al*. Genome degradation in *Brucella ovis* corresponds with narrowing of its host range and tissue tropism. *PLoS ONE.* **4**, e5519 (2009).
15. Mancilla, M., Ulloa, M., López-Goñi, I., Moriyón, I. & María Zárraga, A. Identification of new IS711 insertion sites in *Brucella abortus* field isolates. *BMC Microbiol.* **11**, 176 (2011).
16. Bricker, B. J. & Halling, S. M. Differentiation of *Brucella abortus* bv. 1, 2, and 4, *Brucella melitensis*, *Brucella ovis*, and *Brucella suis* bv. 1 by PCR. *J. Clin. Microbiol*. **32**:2660-2666 (1994).
17. Gyuranecz, M. *et al*. Natural IS711 insertion causing omp31 gene inactivation in *Brucella ovis. J. Vet. Diagn. Invest.* **25**, 234-238 (2013).
18. Altman, D. G. Statistics in medical journals: Developments in the 1980s. *Statist. Med*. **10**, 1897-1913 (1991).
19. Robinson, D. G., Lee, M.-C. & Marx, C. J. OASIS: an automated program for global investigation of bacterial and archaeal insertion sequences. *Nucleic Acids Res.* **40**, 22: e174-e174. (2012).
20. Mahillon, J. & Chandler, M. Insertion Sequences. Microbiol Mol Biol Rev. **62,** (3): 725-774 (1998).

1. Vandecraen, J., Chandler, M., Aertsen, A. & Van Houdt, R. The impact of insertion sequences on bacterial genome plasticity and adaptability. *Crit. Rev. Microbiol.* **43**, 709-730 (2017).
2. Wu, Y., Aandahl, R. Z. & Tanaka, M. M. Dynamics of bacterial insertion sequences: can transposition bursts help the elements persist? *BMC Evol. Biol.* **15**, 288 (2015).
3. Cheung, V. G., et al. "Integration of cytogenetic landmarks into the draft sequence of the human genome." *Nature.* **409**, 6822: 953 (2001).
4. She, X. *et al*. The structure and evolution of centromeric transition regions within the human genome*. Nature.* **430**, 857-864 (2004).
5. Ji, Y. Structure of Chromosomal Duplicons and their Role in Mediating Human Genomic Disorders. *Genome Res.* **10**, 597-610 (2000).
6. Bailey, J. A., Baertsch, R., Kent, W., Haussler, D. & Eichler, E. E. Hotspots of mammalian chromosomal evolution. *Genome Biol.* **5**, R23 (2004).
7. Rajashekara, G., Glasner, J. D., Glover, D. A. & Splitter, G. A. Comparative whole-genome hybridization reveals genomic islands in *Brucella* species. *J. Bacteriol.* **186**, 5040-5051 (2004).

**Authors Contribution Statement:**

S.P. and H.V.B. made the study design and S.P., P.V.B. and M.G. performed the experiments and S.N. prepared figures and S.P. and P.V.B. prepared the manuscript and S.R., B.K. and H.V.B. reviewed and approved the manuscript.

| **Reference Strains** | **Provider** | **No. of strains (n)** | **PCR product size (bp) with species-specific primer** |
| --- | --- | --- | --- |
| *B. abortus* S19 | IVRI* | 1 | 1154 |
| *B. abortus* 544 | IVRI | 1 | 1154 |
| *B. melitensis* 16M | IVRI | 1 | 745 |
| *B. ovis* ATCC 25840 | NVSL** | 1 | 446 |
| *B. suis* 1330 | IVRI | 1 | 290 |
| *B. suis* ATCC 23445 | ATCC*** | 1 | 383 |
| *B. canis* ATCC 23365 | NVSL | 1 | 224 |
| *Pseudomonas aeruginosa* ATCC 27853 | ATCC | 1 | No amplification |
| *P. azotogenesis* NCIM 2075 | NCIM^ƚ^ | 1 | No amplification |
|  |  | | |
| **Species and biovars** | **Host** | | |
| *B. abortus* biovar 1 | Bovine | 16 | 1154 |
| *B. abortus* biovar 2 | Bovine | 12 | 1154 |
| *B. abortus* biovar 3 | Bovine | 9 | 1154 |
| *B. abortus* biovar 4 | Bovine | 8 | 1154 |
| *B. abortus* biovar 5 | Bovine | 2 | 1154 |
| *B. abortus* biovar 6 | Bovine | 4 | 1154 |
| *B. abortus* biovar 7 | Bovine | 2 | 1154 |
| *B. abortus* biovar 9 | Bovine | 1 | 1154 |
| *B. melitensis* biovar 1 | Human | 10 | 745 |
| *B. melitensis* biovar 2 | Human | 6 | 745 |
| *B. melitensis* biovar 3 | Human | 3 | 745 |
| *B. suis* biovar 1 | Human | 2 | 290 |
| *B. suis* biovar 2 | Human | 2 | 290 |
| *B. suis* biovar 3 | Swine | 1 | 383 |
| *B. suis* biovar 4 | Reindeer | 1 | 383 |
| *B. suis* biovar 5 | Rodent | 1 | 383 |
| *Aeromonas hydrophila* isolate DFRL1 | Human | 1 | No amplification |
| *Proteus mirabilis* isolate DFRL1 | Human | 1 | No amplification |
| *Citrobacter freundii* isolate DFRL1 | Human | 1 | No amplification |
| *Yersinia enterocolitica* isolate DFRL1 | Human | 1 | No amplification |

* IVRI – Indian Veterinary Research Institute, Bareilly, Uttar Pradesh, India

** NVSL – National Veterinary Services Laboratories, Ames, Iowa

*** ATCC – American Type Culture Collection, USA

^ƚ^ NCIM – National Collection of Industrial Microorganisms, Pune, India

**Table 1. Bacterial strains used in this study and the resultant PCR amplicon size (bp) with species-specific primer pair in monoplex PCR**

| **Primer ID** | **Sequence (5’ to 3’)** | **DNA target** | **Amplicon**  **size (bp)** | |
| --- | --- | --- | --- | --- |
|  |  |  | **Specific** | **Rescue** |
| BAA13334_I00002f | TGTATCGTACTTCATACTCC | Conserved hypothetical protein | 1154 | 1478 |
| BAA13334_I00002r | GCCGCAGAGACACGACAGAA |  |  |  |
| BMEA_A1673f | TCTCCTTGATTATGGTGCGA | Outer membrane protein, gene *Omp31* | 745 | 977 |
| BMEA_A1673r | GACGTCTTGACCTTACCAT |  |  |  |
| BOV_1671f | GTTCCGGGCGACGCCAT | Phosphoglycerate kinase, gene *pgk* | 446 | 606 |
| BOV_1671r | GCAGCTTGGAGCATTATCC |  |  |  |
| BS1330_II0128f | AGTTGCCCCGCCTTCTGGAG | Flagellum-specific ATP synthase, gene *fliI* | 290 | 383 |
| BS1330_II0128r | TTCATAAATACGCGGCACAA |  |  |  |
| BCAN_A0740f | TTTTTCTGTCCACCATTTATC | GCN5-related N-acetyltransferase | 224 | 521 |
| BCAN_A0740r | ACAATAAAACAGTAGAGCCG |  |  |  |
| IAC-FP | *TGTATCGTACTTCATACTCC***AGCTGAATGAAGCCATACCA** | pUC19 | 847 | |
| IAC-RP | *GCCGCAGAGACACGACAGAA* **AACCCGGTAAGACACGAC** |  |  |  |

**Table 2: List of primers used in multiplex PCR assay**

**Figure Legends**

**Figure 1:** Scheme for mining *Brucella* species-specific nucleotide sequences.

**Figure 2:** Pictorial illustration of the primer designing strategy using whole genome sequence for specific detection of *B. abortus, B. melitensis, B. ovis, B. suis* and *B. canis*.

**Figure 3: Agarose gel electrophoretic analysis of monoplex PCR format for specific detection of *B. abortus, B. melitensis, B. ovis, B. suis* and *B. canis*.** Lane 1 and 7. 50 bp DNA ladder; Lane 2. *B. abortus* specific amplicon (1154 bp) with *B. abortus* specific primer; Lane 3. *B. melitensis* specific amplicon (745 bp) with *B. melitensis* specific primer; Lane 4. *B. ovis* specific amplicon (446 bp) with *B. ovis* specific primer; Lane 5. *B. suis* specific amplicon (290 bp) with *B. suis* specific primer; Lane 6. *B. canis* specific amplicon (224 bp) with *B. canis* specific primer; Lane 8. *B. abortus* rescue amplicon (1478 bp) with primers of the present study except *B. abortus* specific primer; Lane 9. *B. melitensis* rescue amplicon (977 bp) with primers of the present study except *B. melitensis* specific primer; Lane 10. *B. ovis* rescue amplicon (606 bp) with primers of the present study except *B. ovis* specific primer; Lane 11. *B. suis* rescue amplicon (383 bp) with primers of the present study except *B. suis* specific primer; Lane 12. *B. canis* rescue amplicon (521 bp) with primers of the present study except *B. canis* specific primer.

**Figure 4: Agarose gel electrophoretic analysis of PCR assay using *B. abortus, B. melitensis, B. ovis, B. suis* and *B. canis* genomic DNA as template and one pair of primer per reaction.** Lane 1 and 7. 50 bp DNA ladder; Lane 2. Specific and rescue amplicon resulted from *B. abortus* specific primer pair (1154 bp and 1478 bp); Lane 3. Specific and rescue amplicon resulted from *B. melitensis* specific primer pair (745 bp and 977 bp); Lane 4. Specific and rescue amplicon resulted from *B. ovis* specific primer pair (446 bp and 606 bp); Lane 5. Specific and rescue amplicon resulted from *B. suis* specific primer pair (290 bp and 383 bp); Lane 6. Specific and rescue amplicon resulted from *B. canis* specific primer pair (224 bp and 521 bp).

**Figure 5:** **Agarose gel electrophoretic analysis of multiplex PCR assay optimized by incorporation of an internal amplification control (847 bp). All five novel primer pairs were involved in the PCR reaction.** Lane 1 and 10. 50 bp DNA ladder; Lane 2 and 9. PCR using *B. abortus, B. melitensis, B. ovis, B. suis* and *B. canis* genomic DNA as template DNA; Lane 3. PCR using *B. abortus* genomic DNA as template DNA; Lane 4. PCR using *B. melitensis* genomic DNA as template DNA; Lane 5. PCR using *B. ovis* genomic DNA as template DNA; Lane 6. PCR using *B. suis* genomic DNA as template DNA; Lane 7. PCR using *B. canis* genomic DNA as template DNA; Lane 8. PCR without any genomic DNA.

**Figure 6:** Figure illustrating the interpretation of multiplex PCR formats for specific detection of *Brucella* species. Lane1: Specific PCR amplicons of all 5 *Brucella* species are observed for species identification and differentiation; Lane 2: Specific PCR amplicon of 1154 bp indicates presence of *B. abortus*; Lane 3: Specific PCR amplicon of 745 bp indicates presence of *B. melitensis*; Lane 4: Specific PCR amplicon of 446 bp indicates presence of *B. ovis*; Lane 5: Specific PCR amplicon of 290 bp indicates presence of *B. suis*; Lane 6: Specific PCR amplicon of 224 bp indicates presence of *B. canis*; Lane 7: Specific PCR amplicons of 1154 bp and 745 bp indicates presence of *B. abortus* and *B. melitensis*; Lane 8: Specific PCR amplicons of 1154 bp and 446 bp indicates presence of *B. abortus* and *B. ovis*; Lane 9: Specific PCR amplicons of 446 bp and 290 bp indicates presence of *B. ovis* and *B. suis*; Lane 10: Specific PCR amplicons of 290 bp and 224 bp indicates presence of *B. suis* and *B. canis*; Lane 11: Specific PCR amplicons of 1154 bp, 745 bp and 446 bp indicates presence of *B. abortus, B. melitensi* and *B. ovis*; Lane 12: PCR amplicon of 847 bp only indicates that the sample is negative for contamination with any of the five *Brucella* species.

For instance, multiplex PCR reaction is performed with a test sample contaminated with all 5 *Brucella* species namely *B. abortus, B. melitensis, B. suis, B. canis* and *B. ovis,* the result will be as follows: (1) *B. abortus* specific primer pair yields amplicon of 1154 bp with *B. abortus* and 1478 bp with other 4 species; (2) *B. melitensis* specific primer pair 745 bp with *B. melitensis* and yields 977 bp with other 4 species; (3) *B. suis* specific primer pair yields 290 bp with *B. suis* and 383 bp with other 4 species; (4) *B. canis* specific primer pair yields 224 bp with *B. canis* and 521 bp with other 4 species; (5) *B. ovis* specific primer pair yields 446 bp with *B. ovis* and 606 bp with rest 4 species and (6) IAC specific primer yields 847 bp amplification with IAC DNA.
