# Supplementary figures and images for: Genome-wide unique insertion sequences among five Brucella species and demonstration of differential identification of Brucella by multiplex PCR assay
Source: Sci Rep. 2020 Apr 14;10:6368. doi: 10.1038/s41598-020-62472-3 (PMC7156498; doi:10.1038/s41598-020-62472-3)

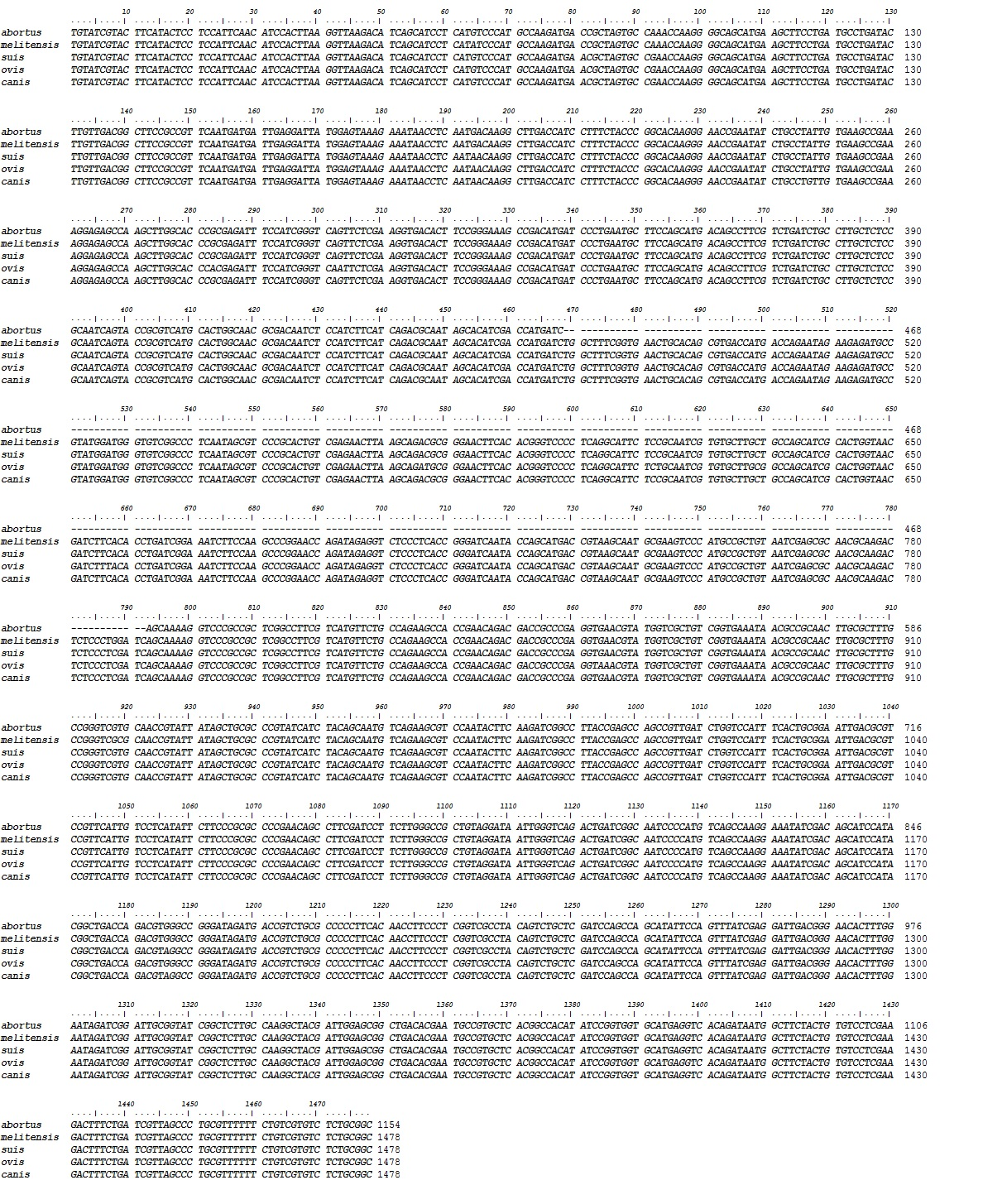

Supplement: Supplementary file 2 — Supplementary Dataset. [file 41598_2020_62472_MOESM2_ESM.zip › Supplementary Dataset/Supplementary_Figure_1.tif]

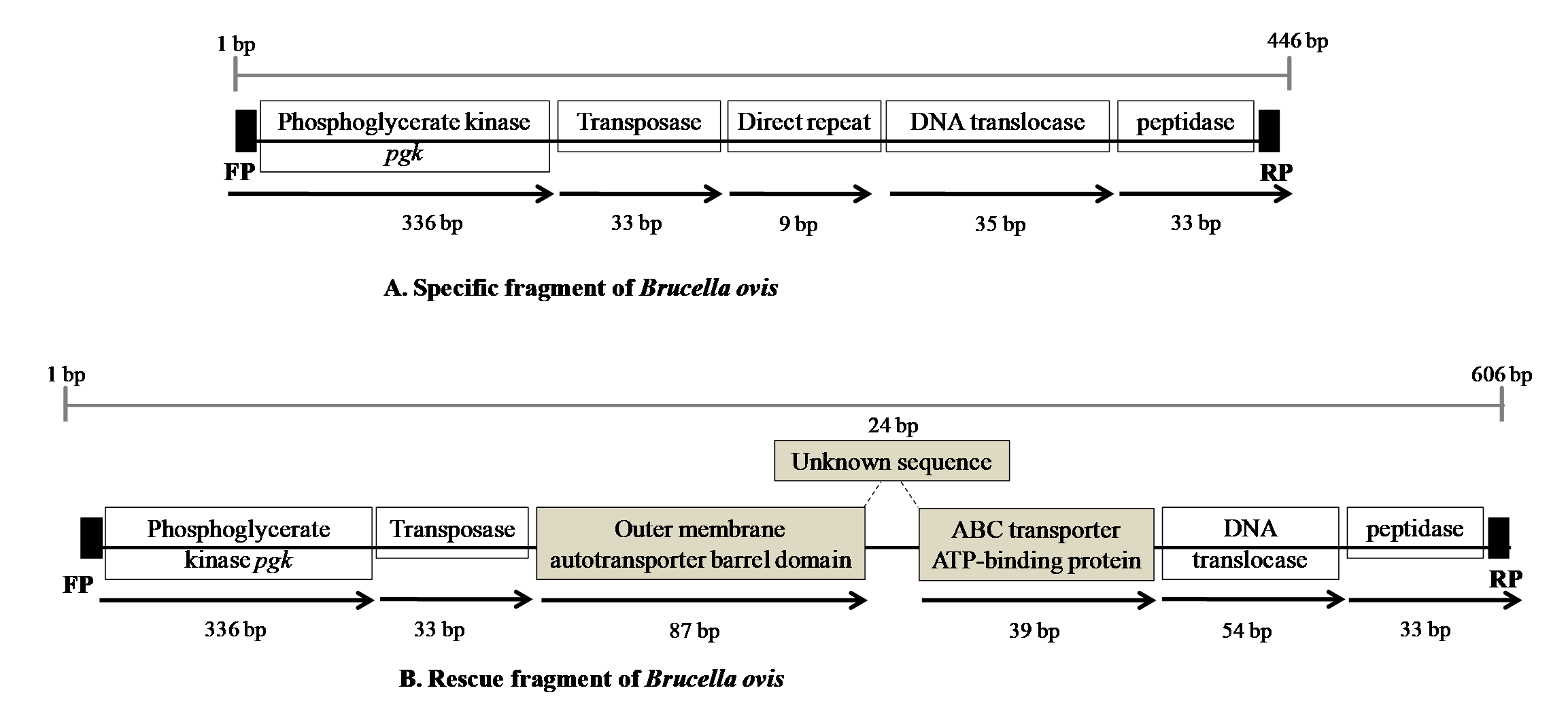

Supplement: Supplementary file 2 — Supplementary Dataset. [file 41598_2020_62472_MOESM2_ESM.zip › Supplementary Dataset/Supplementary_Figure_10.tif]

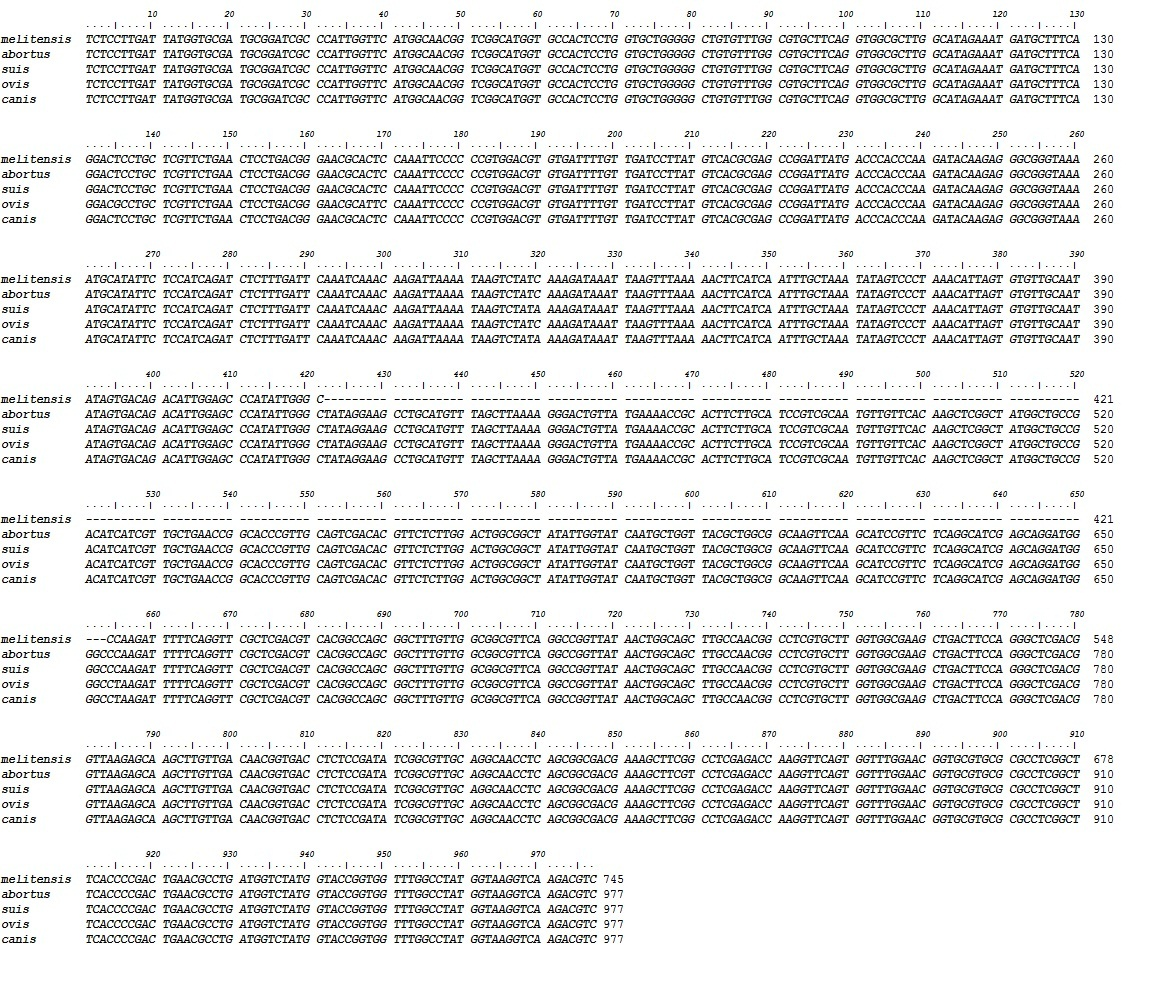

Supplement: Supplementary file 2 — Supplementary Dataset. [file 41598_2020_62472_MOESM2_ESM.zip › Supplementary Dataset/Supplementary_Figure_2.tif]

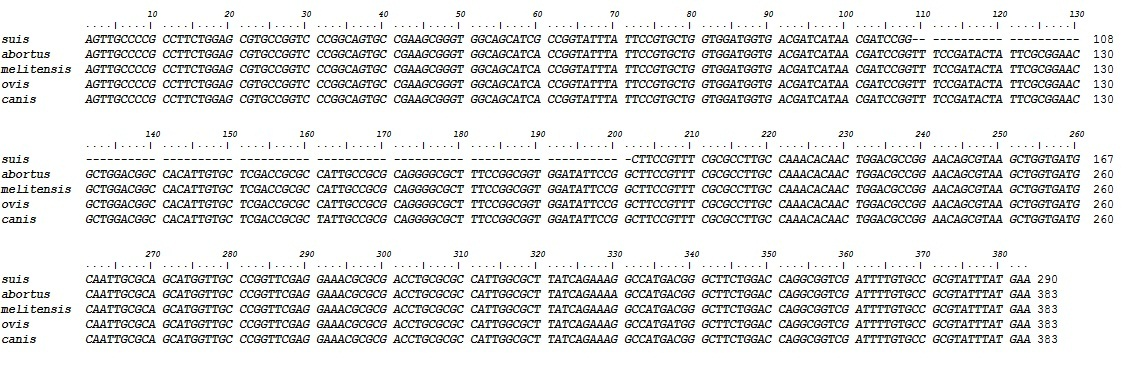

Supplement: Supplementary file 2 — Supplementary Dataset. [file 41598_2020_62472_MOESM2_ESM.zip › Supplementary Dataset/Supplementary_Figure_3.tif]

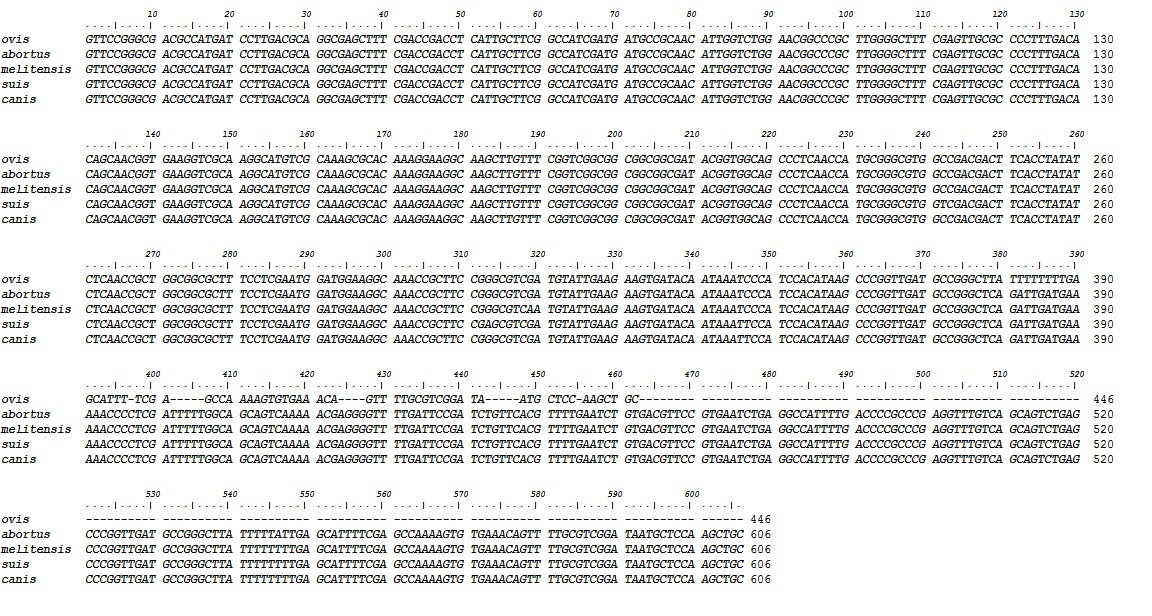

Supplement: Supplementary file 2 — Supplementary Dataset. [file 41598_2020_62472_MOESM2_ESM.zip › Supplementary Dataset/Supplementary_Figure_4.tif]

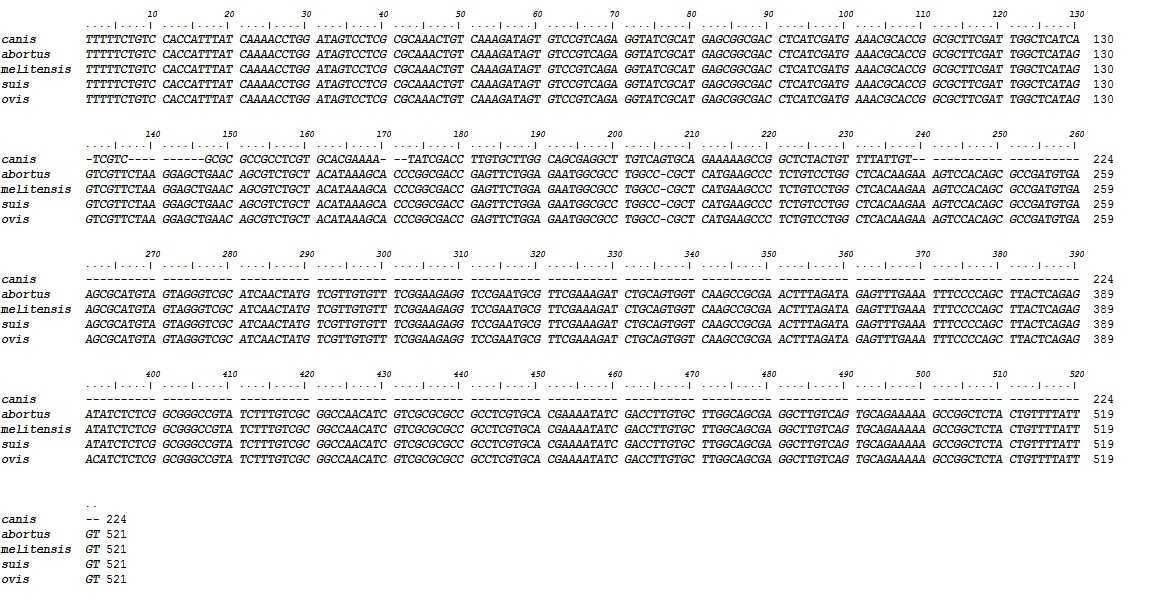

Supplement: Supplementary file 2 — Supplementary Dataset. [file 41598_2020_62472_MOESM2_ESM.zip › Supplementary Dataset/Supplementary_Figure_5.tif]

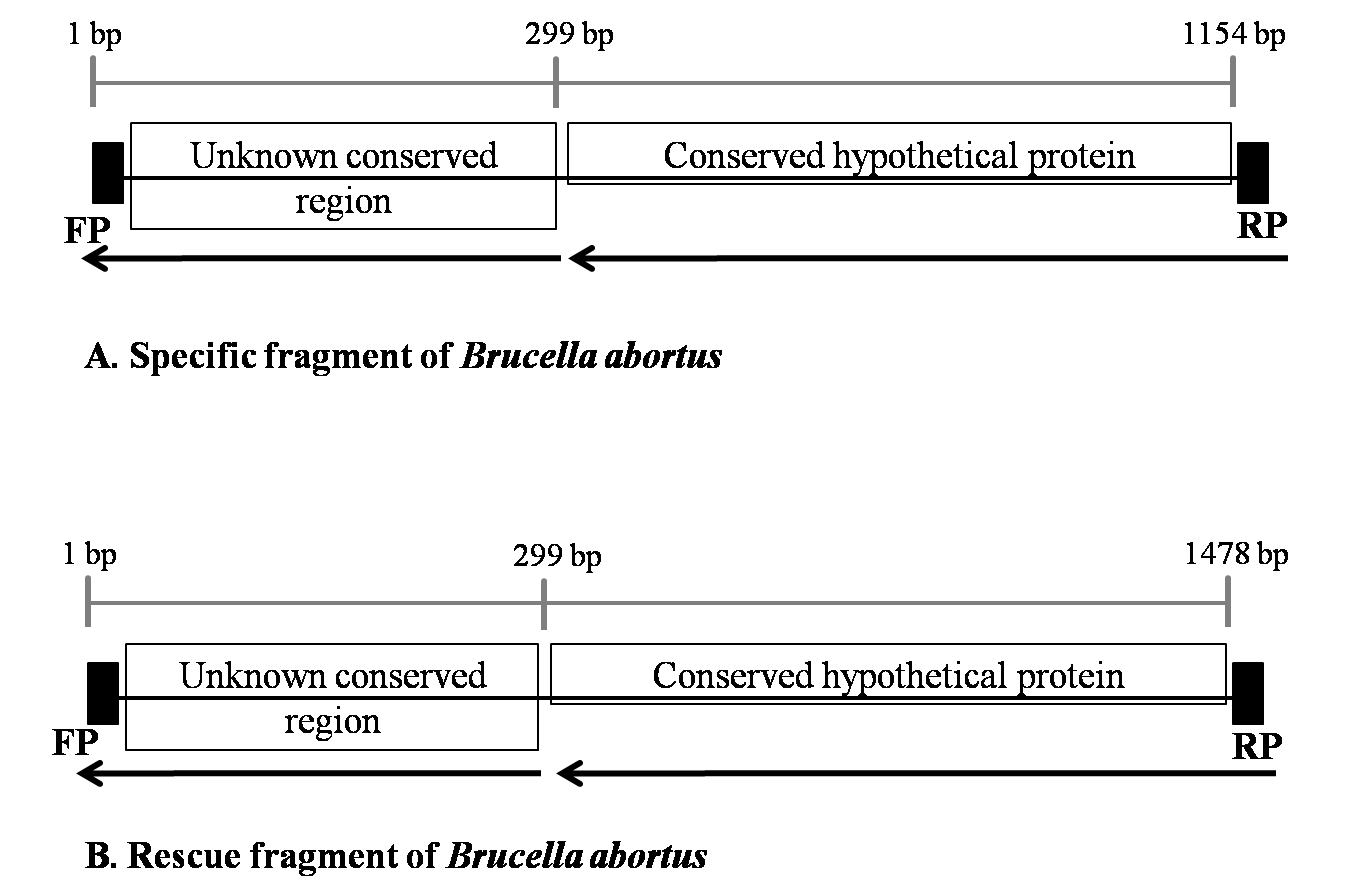

Supplement: Supplementary file 2 — Supplementary Dataset. [file 41598_2020_62472_MOESM2_ESM.zip › Supplementary Dataset/Supplementary_Figure_6.tif]

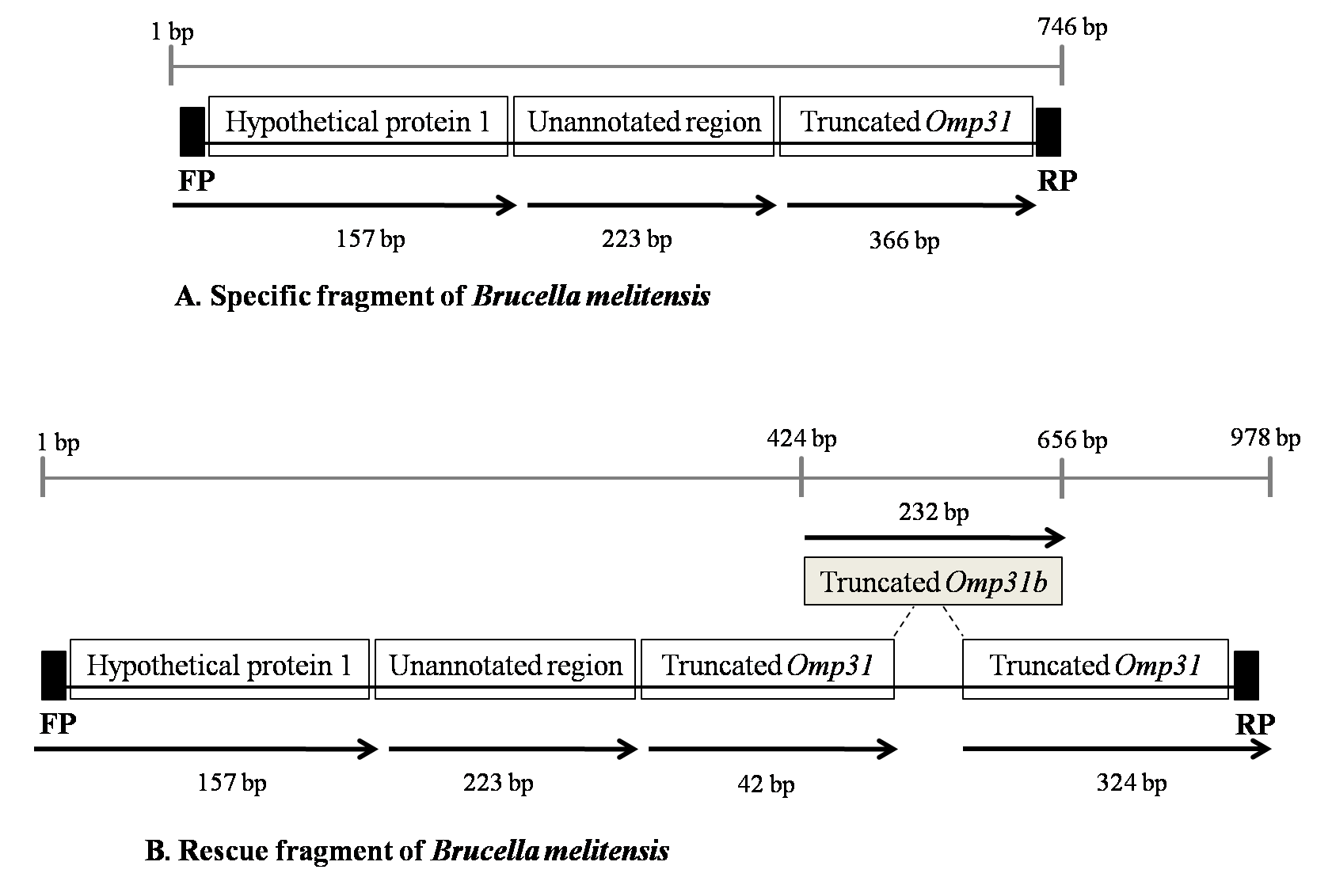

Supplement: Supplementary file 2 — Supplementary Dataset. [file 41598_2020_62472_MOESM2_ESM.zip › Supplementary Dataset/Supplementary_Figure_7.tif]

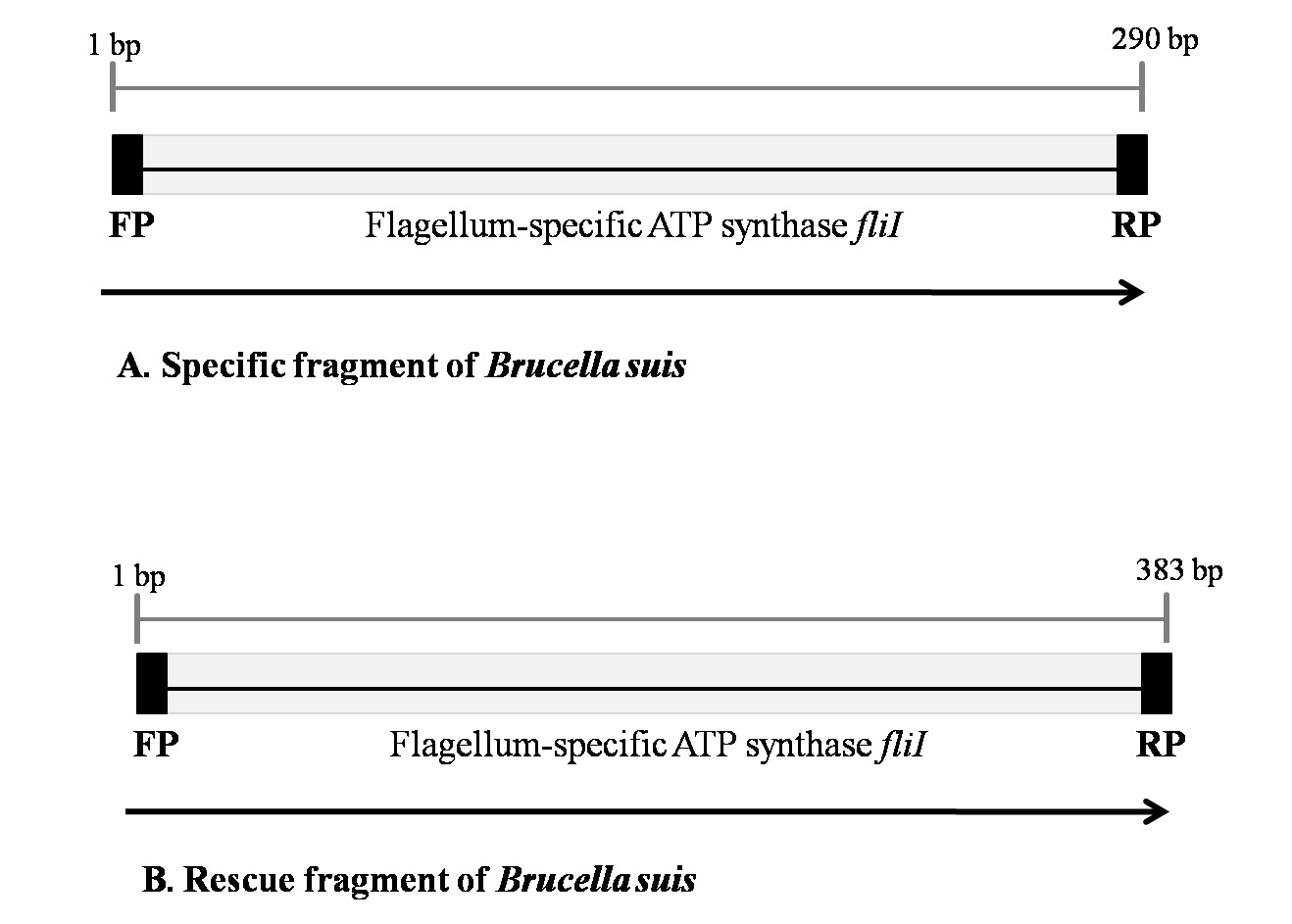

Supplement: Supplementary file 2 — Supplementary Dataset. [file 41598_2020_62472_MOESM2_ESM.zip › Supplementary Dataset/Supplementary_Figure_8.tif]

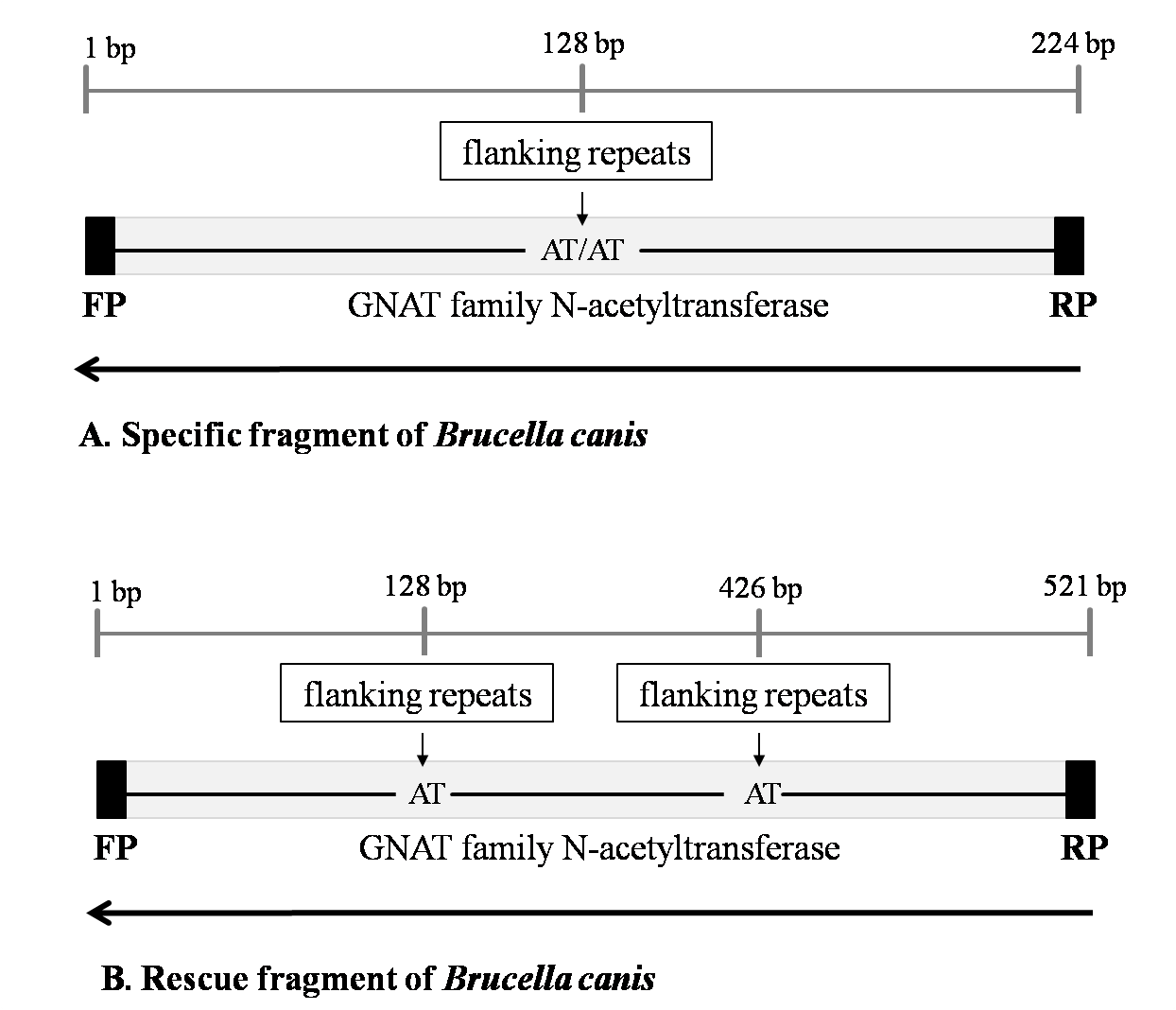

Supplement: Supplementary file 2 — Supplementary Dataset. [file 41598_2020_62472_MOESM2_ESM.zip › Supplementary Dataset/Supplementary_Figure_9.tif]
